# Intrathecal injection of bone marrow stromal cells attenuates neuropathic pain via inhibition of P2X_4_R in spinal cord microglia

**DOI:** 10.1186/s12974-019-1631-0

**Published:** 2019-12-17

**Authors:** Yongbo Teng, Yang Zhang, Shouwei Yue, Huanwen Chen, Yujuan Qu, Hui Wei, Xiaofeng Jia

**Affiliations:** 1 Department of Physical Medicine & Rehabilitation, Qilu Hospital, Medical School of Shandong University, Jinan, China; 2 Department of Neurosurgery, University of Maryland School of Medicine, Baltimore, MD 21201 USA; 3 Department of Orthopedics, University of Maryland School of Medicine, Baltimore, MD 21201 USA; 4 Department of Anatomy Neurobiology, University of Maryland School of Medicine, Baltimore, MD 21201 USA; 5 Department of Biomedical Engineering, Johns Hopkins University School of Medicine, Baltimore, MD 21205 USA; 6 Department of Anesthesiology and Critical Care Medicine, Johns Hopkins University School of Medicine, Baltimore, MD 21205 USA

**Keywords:** Neuropathic pain, Bone marrow stromal cells, P2X_4_R, TRPV4

## Abstract

**Background:**

Neuropathic pain is one of the most debilitating of all chronic pain syndromes. Intrathecal (i.t.) bone marrow stromal cell (BMSC) injections have a favorable safety profile; however, results have been inconsistent, and complete understanding of how BMSCs affect neuropathic pain remains elusive.

**Methods:**

We evaluated the analgesic effect of BMSCs on neuropathic pain in a chronic compression of the dorsal root ganglion (CCD) model. We analyzed the effect of BMSCs on microglia reactivity and expression of purinergic receptor P2X_4_ (P2X_4_R). Furthermore, we assessed the effect of BMSCs on the expression of transient receptor potential vanilloid 4 (TRPV4), a key molecule in the pathogenesis of neuropathic pain, in dorsal root ganglion (DRG) neurons.

**Results:**

I.t. BMSC transiently but significantly ameliorated neuropathic pain behavior (37.6% reduction for 2 days). We found no evidence of BMSC infiltration into the spinal cord parenchyma or DRGs, and we also demonstrated that intrathecal injection of BMSC-lysates provides similar relief. These findings suggest that the analgesic effects of i.t. BMSC were largely due to the release of BMSC-derived factors into the intrathecal space. Mechanistically, we found that while i.t. BMSCs did not change TRPV4 expression in DRG neurons, there was a significant reduction of P2X_4_R expression in the spinal cord microglia. BMSC-lysate also reduced P2X_4_R expression in activated microglia in vitro. Coadministration of additional pharmacological interventions targeting P2X_4_R confirmed that modulation of P2X_4_R might be a key mechanism for the analgesic effects of i.t. BMSC.

**Conclusion:**

Altogether, our results suggest that i.t. BMSC is an effective and safe treatment of neuropathic pain and provides novel evidence that BMSC’s analgesic effects are largely mediated by the release of BMSC-derived factors resulting in microglial P2X_4_R downregulation.

## Introduction

Neuropathic pain is a common and debilitating manifestation of various peripheral nerves diseases [1, 2]. Despite the obvious need for effective interventions, neuropathic pain remains a pressing clinical problem owing to the limited efficacy of available drugs and poor understanding of its pathophysiology [3, 4]. Bone marrow stromal cells (BMSCs), known to exhibit immunomodulatory properties [5, 6] and attenuate tissue damage caused by the excessive inflammation [5, 7], are especially promising as a candidate for novel therapies [4, 7–10]. Intravenous and local injections (into ventricle space or dorsal root ganglion, DRG) of BMSCs have been shown to ameliorated pain hypersensitivity in various models of neuropathic pain [8–11]. However, while clinical trials of BMSCs have shown no major adverse events over the past 10 years [7, 12], systemic BMSC therapy can lead to potentially lethal microemboli of BMSCs into the pulmonary circulation [10]. Moreover, local injection of BMSCs can also result in traumatic nerve damage [13]. Given these concerns with intravenous and local administration of BMSCs, the safety and efficacy of intrathecal (i.t.) delivery of BMSC have been explored and investigated. To date, results have suggested that i.t. delivery of BMSC into thoracic and lumbar spinal cerebrospinal fluid (CSF) features a favorable safety profile as it has higher tissue-specificity, less systemic side effects, and smaller dose requirements as measured by the total number of implanted cells [9, 14, 15]. However, in the context of neuropathic pain, efficacy results of i.t. BMSC have been variable, ranging from a significant improvement in symptoms for several weeks [9] to negligible behavioral changes [14, 15]. These conflicting results might be due in part to the lack of a thorough understanding of how BMSCs act to alleviate neuropathic pain, thereby resulting in sub-optimal experimental designs to investigate its efficacy. Thus, further investigation into BMSCs’ mechanism is needed to fully optimize its analgesic application.

The mechanisms of neuropathic pain are immensely complex, involving both structural and functional changes throughout the nociceptive pathways, spanning the entire nervous system from the site of peripheral nerve injury to the DRG, spinal cord, and brain [16, 17]. The immune system plays a major role in neuropathic pain, with multiple reports suggesting that spinal cord microglia, the principal central nervous system immune cells responsible for surveillance, support, protection, and restoration of tissue integrity [18], interact extensively with spinal cord neurons and are critical for initiation and chronicity of hypersensitivity associated with neuropathic pain [17–23]. More specifically, a subtype of ionotropic ATP receptor (purinergic receptor P2X4, aka P2X_4_R) expressed in microglia is thought to play an especially important role in the pathogenesis of pain hypersensitivity [24, 25]. Reports have demonstrated that stimulation of microglia P2X_4_R initiates core pain signaling pathways, leading to phenotypic switches in the response properties of nociceptive (pain-transmitting) neurons in lamina I [3, 20, 26]. Thus, interventions targeting spinal microglia and aborting microglia-induced local neurobiological alterations may hold the key to more effective therapies for neuropathic pain [21, 23, 26–28].

While both BMSCs and microglia are known to impact neuropathic pain, whether the mechanism of BMSC therapy involves microglia regulation remains largely unknown. We hypothesized that BMSC transplantation attenuates neuropathic pain induced by chronic compression of dorsal root ganglion (CCD), and that BMSC’s effect on neuropathic pain is associated with microglia. To explore the mechanism of and provide critical insight into BMSC therapy for optimization and clinical translation, we investigated the analgesic effect of i.t. injection of BMSC in a CCD model in rats, studied the microglial reaction in response to CCD and BMSC, and explored interactions between BMSCs and microglia.

## Methods

### Animals and surgical procedure

**Fig. 1:**
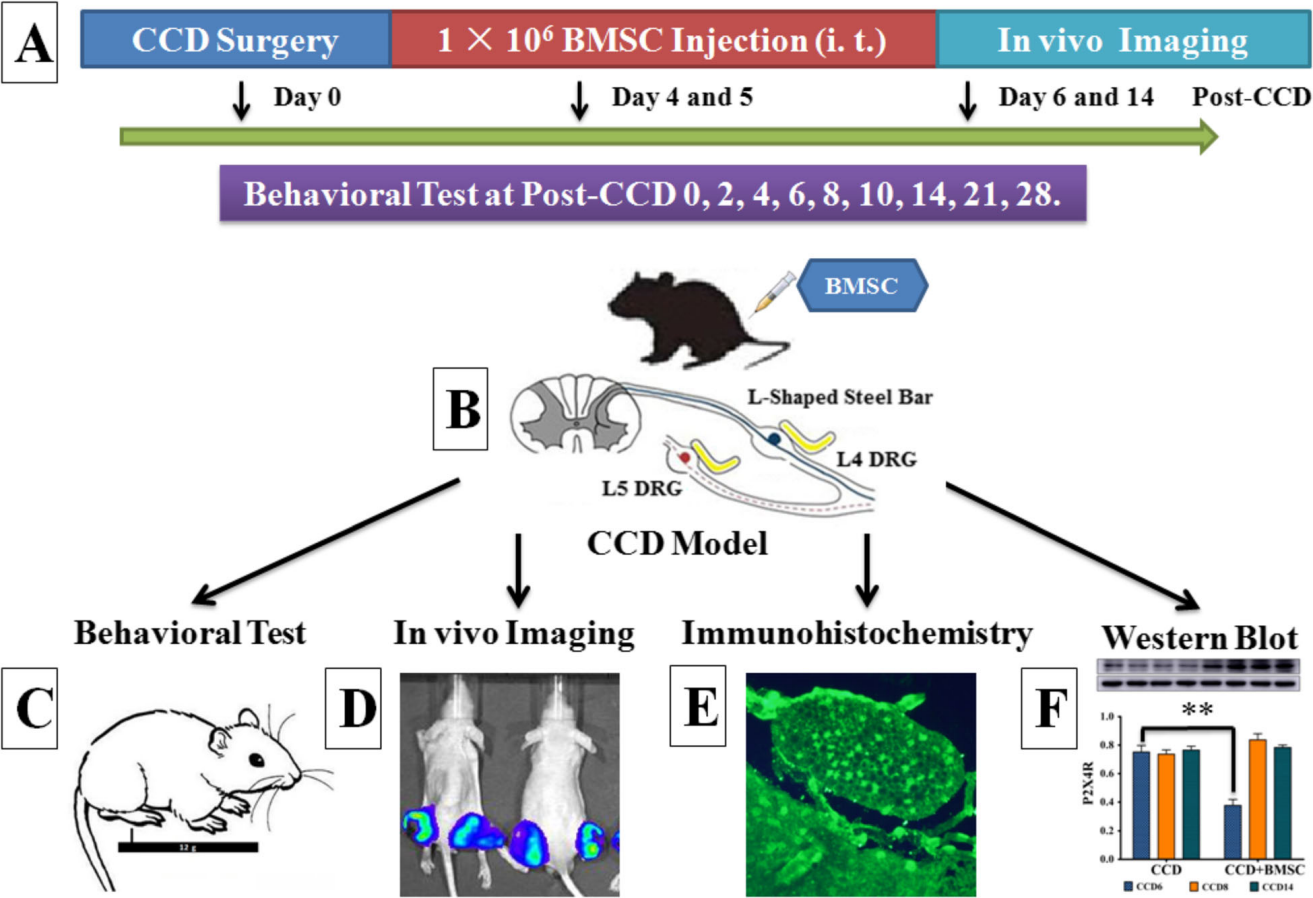
Experimental design for in vivo experiments. BMSC, bone marrow stromal cells; CCD, chronic compression of dorsal root ganglion. (**a**) Animal experiment design and procedures; (**b**) BMSCs were injected into CCD rats to mitigate neuropathic pain. (**c**–**f**) Multimodal evaluations of neuropathic pain after BMSC transplantation including behavioral tests (**c**), in vivo imaging (**d**), immunohistochemistry (**e**), and Western blot (**f**)

The overall design for experimental procedures is outlined in Fig. 1. Male Wistar rats (bodyweight 180–200 g; Shandong University Lab Animal Center, Jinan, China) were housed individually in plastic bottomed cages containing wood shaving as bedding. The room was temperature-controlled (23 ± 2 °C), with a maintained relative humidity of 40–60% and a 12:12 h light-dark cycle (lights on at 07:00 AM). Rats were fed a standard laboratory diet of rat chow pallets; food and water were available ad libitum. All animal procedures were reviewed and approved by the Chinese Institutional Animal Care Committee of Shandong University and were carried out in accordance with the Helsinki declaration. All efforts were made to minimize the number of animals used and their suffering.

The CCD model was established with intervertebral foramen stenosis, which is achieved via an approach as previously described [29–31]. Briefly, under pentobarbital sodium anesthesia (50 mg/kg i.p.), the right transverse process and intervertebral foramina of L4 and L5 were exposed unilaterally. Two stainless steel L-shaped rods (0.63 mm in diameter and 4 mm in length) were inserted into each foramen. The incision site was sutured in layers and penicillin was injected intraperitoneally to prevent infection. The sham group underwent the same surgical procedure with the exception of rod insertion. Correct placement of the rods was confirmed during DRG harvesting. All surgeries were performed by the same surgeon (YBT) to ensure consistency and the total procedural time was kept under 2 min for each animal.

### Behavioral testing

Walk gait pattern was assessed as an index of motor function [32] on postoperative day 2 for all animals (*n* = 80). Only rats scoring 1, which indicates a normal gait, were used in following experimental procedures and other animals (*n* = 5) were euthanized. For nociceptive behavior testing, animals were placed in individual Plexiglas cubicles (15 cm L × 10 cm W × 25 cm H) on a wire mesh platform and habituated to the surroundings for 30 min. Mechanical allodynia was measured as the number of hind paw withdrawals elicited by a defined innocuous mechanical stimulus (12 g von Frey filaments) [33] before surgery and on days 2, 4, 6, 8, 10, 14, 21, and 28 post-surgery. In each testing session, rats were subjected to three rounds of 10 tactile stimulations with at least 10 min between rounds. Licking was also measured during the mechanical stimulation of the hind paw, which has been cited as hyperalgesia behavior and is associated with an aversive experience in the context of neuropathic pain [22]. Motor coordination was measured by accelerating rotarod (Yuyan Instruments Co., Ltd., Shanghai China) before and after i.t. delivery of BMSC. For all behavioral analyses, the observer was blinded to the injury type and treatment conditions of the rats.

### Cell culture

Rat BMSC isolated from the bone marrow of 4-week-old donor Wistar rats were obtained from ScienCell Research Laboratories (Lot No. 8567). According to the vendor, these were frozen at the fourth passage, and express flow-cytometry cell surface marker CD29, CD44, CD90, and CD106 (> 70%) but negative for CD11b, CD34, and CD45 (< 5%). Their ability to differentiate into osteocytes, adipocytes, and chondrocytes has been experimentally validated. We, therefore, used the cells for subsequent experiments without further characterization. Cells were cultured in low glucose Dulbecco’s modified Eagle’s medium containing 1 g/L glucose (DMEM-LG, Hyclone, Beijing, China) supplemented with 10% fetal bovine serum (FBS, Gibco, Australia) and antibiotic-antimycotic mixture (100 IU/mL penicillin, 16 μg/mL streptomycin, and 10 μg/mL amphotericin B), and were maintained at 37 °C in a 5% CO_2_ incubator (SANYO Electric Co., Ltd., Japan) in fully humidified air. The medium was refreshed every 2 days, and adherent BMSCs were grown to 90% confluence and passage 6 was used for in vivo study.

Fibroblast cultures were prepared from the dorsal skin of postnatal day 1 Wistar rats as described previously [34]. The cell pellet was resuspended and cultured at 37 °C under 5% CO_2_ in Dulbecco’s modified Eagle’s medium (DMEM; Gibco, USA) with L-glutamine (2 mM), 10% fetal bovine serum (FBS; Gibco, USA), penicillin (100 IU/mL), and streptomycin (100 mg/mL) (Thermo, USA). Cell passage 4 was used for the following experiments.

Rat primary cultured microglia were prepared from postnatal (P1–P3) Wistar rat for 10–14 days as described previously [35]. Microglia were separated by gentle shakes of the mixed glial culture at 14–15 days in vitro and transferred to appropriate dishes coated with or without fibronectin (FN, 10 μg/mL; Sigma, St. Louis). Primary microglia were incubated with BMSC (one million/dish) lysate or PBS for 24 h at 37 °C and then used for further experiments and analyses.

### Intrathecal transplantation of cells

Previous studies have shown that intrathecal catheterization causes an upregulation of spinal cytokine IL-6 [33], so, we adopted direct percutaneous i.t. injections for the transplantation of cells. Under isoflurane anesthesia (2% supplied with oxygen), a HAMILTON microsyringe (25 μL) was slowly advanced through the L4–L5 interspace until it reached the subarachnoid space. Needle placement in the subarachnoid space was confirmed by a tail flick.

For cell tracking, BMSCs were first labeled by chloromrthylbenzamido (CelltrackerTM CM-DiI; C7000). Cell numbers were counted by a cell counter and cell concentration was adjusted to 10^6^/15 μL. The dose of BMSC was determined based upon prior investigations and permits the delivery of a large number of cells in a minimal volume without cell clustering [15]. One dose of cells (15 μL) was slowly injected within 5 min at an interval rate of 3 μL/min. For the control group, skin fibroblasts were delivered instead of BMSC with identical dose and delivery. Injections were performed twice on postoperative days 4 and 5.

For microglia investigations, primary microglial cells were cultured with or without BMSC-lysate for 24 h. Immediately prior to administration, cells were stimulated with ATP at 50 μM and incubated for 1 h at 37 °C [22, 25]. Microglial cells with the supernatant (10,000 cells/10 μL) were then injected intrathecally to normal rats, and behavioral analyses were conducted directly before and 5 h after injection.

### Tracking and relocalization of BMSC

Tracking of BMSCs was performed using the IVIS imaging system (IVIS; Caliper Life Sciences). On 6 and 14 days after CCD (or 1 and 9 days after the last i.t. injection of BMSC), animals were anesthetized by isoflurane. To facilitate the detection of grafted cells, the skin, spinous process, and vertebral plate at T12–S1 level, which collectively shelter the effective detection of CM-DiI signals, were removed. A grayscale reference image was taken of the position of rats prior to assessing fluorescence intensity. The grayscale photographic and fluorescent images were superimposed using the Living Image V 4.2. Pseudocolor images representing the fluorescence signal intensity (crimson being the least intense and yellow being the most intense). To detect the re-localization of injected BMSC, spinal cords and DRGs were embedded in OCT compound; 8-um-thick sections were cut with a cryostat at − 20 °C (light-tight). The slices of spinal cords and DRGs were counterstain with DAPI for nuclei and then analyzed under a fluorescence microscope directly.

### Tissue collection

At predetermined end times, rats were deeply anesthetized with pentobarbital sodium (Nembutal, 50 mg/kg i.p.) and perfused transcardially with cooled (4 °C) PBS followed by preparation for either immunohistochemistry (IHC) or Western blot. Immediately after perfusion, the spinous process and vertebral plate were removed, and the spinal cord was isolated carefully and post-fixed overnight [32]. For immunohistochemistry, the tissues were dehydrated and paraffin infused. For Western blot, the lumbar enlargement was isolated, and ipsilateral and contralateral sides were separated using the ventral fissure as a reference. Spinal cords and DRGs were stored in microtubes at − 80 °C until analysis.

### Western blot

Western blot was performed using standard protocols as previously described [32]. Briefly, the protein of spinal cords and cultured microglia were separated on a 10% SDS-polyacrylamide gel electrophoresis gel, blotted to a nitrocellulose membrane (0.45um), and incubated overnight at 4 °C with rabbit anti- transient receptor potential vanilloid 4 (TRPV4) antibodies (1:700, Abcam, Shanghai, China) and rabbit anti-P2X_4_R antibodies (1:800, Abcam, Shanghai, China). The membrane was washed with TBST and incubated with anti-rabbit IgG (1:8000, ZSGB-BIO, Beijing, China); immunoreactivity was detected using enhanced chemiluminescence (Millipore, Beijing, China). GAPDH served as a loading control. Band intensities were quantified by Image J software [36] and the protein levels were expressed as a ratio of the intensity of the detected band over the respective controls.

### Immunohistochemistry

A series of 5-μm paraffin sections were cut using a rotary microtome. Staining was performed by the standard protocol as previously described [32, 36]. After washing, sections were immunolabeled with the primary antibodies of rabbit polyclonal P2X_4_R (1:300, Abcam, Shanghai, China), mouse monoclonal OX42 (1:400, Abcam, Shanghai, China), rabbit polyclonal TRPV4 (1:200, Abcam, Shanghai, China), and BSA for negative control. The images of the labeled sections were captured and examined using an Olympus-DP72 automated research microscope and analyzed using IPP.6.

### Statistical analyses

All data are presented as mean ± SEM and differences between groups were examined for statistical significance using generalized linear models repeated measures followed by LSD post hoc test correcting for multiple comparisons, one-way factorial analysis of variance (ANOVA), or Student’s *t* test. Significant differences are indicated if *P* values were below 0.05. The above tests were conducted using SPSS software version 17.0 (SPSS, Chicago, IL, USA). GraphPad Prism software version 5.00 (GraphPad Software, San Diego, CA, USA) was used for data representation.

## Results

### Intrathecal delivery of BMSC leads to a strong and transient reduction of CCD-induced mechanical allodynia

**Fig. 2:**
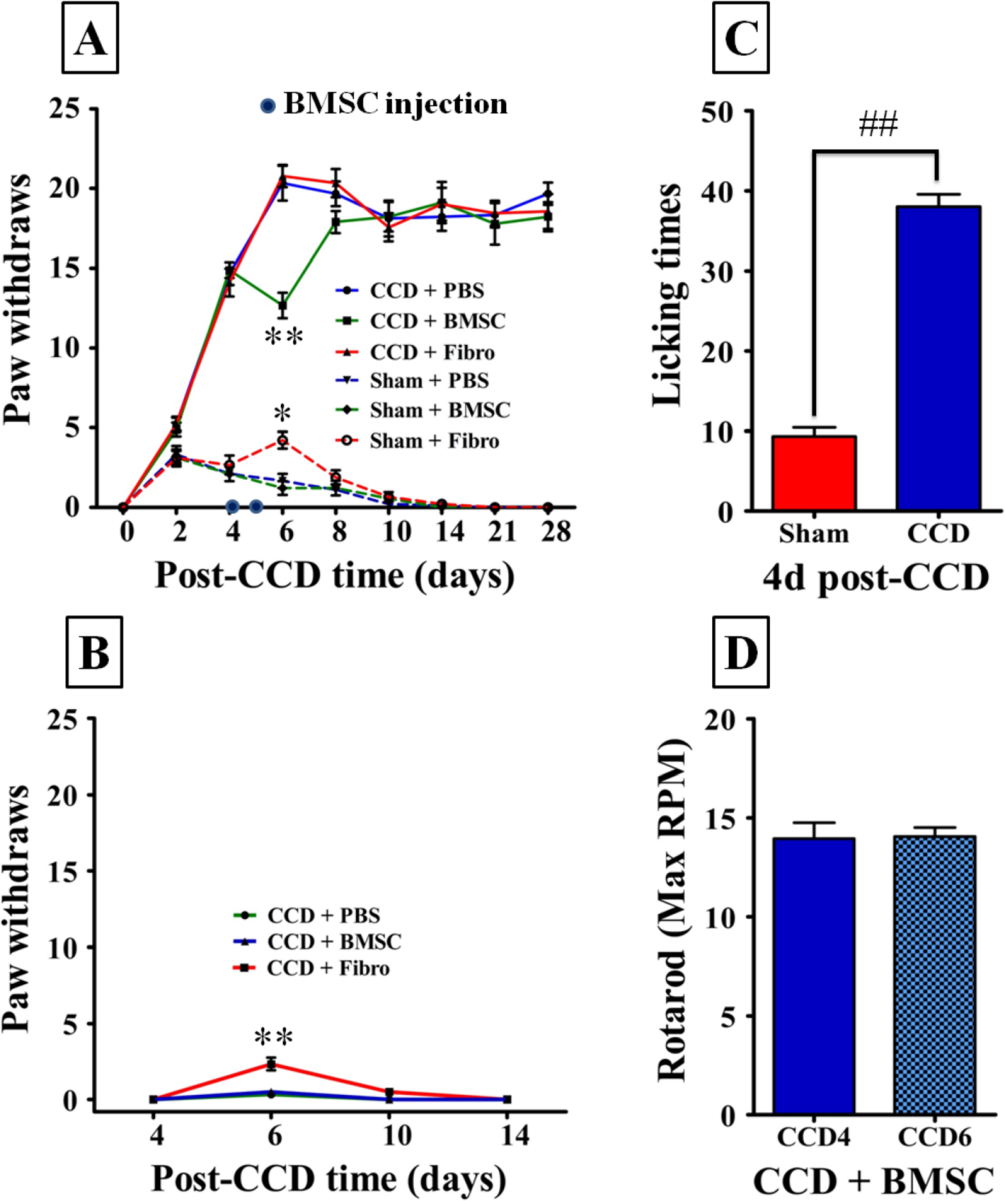
Intrathecal delivery of BMSCs but not fibroblasts transiently attenuated mechanical allodynia. **a** CCD induced significant mechanical allodynia ipsilaterally (measured by foot lift response frequency to stimulation with 12 g von Frey filament), and sham surgery produced transient hypersensibility compared to the baseline value. BMSCs (i.t.) induced a significant (**p < 0.01) reduction of mechanical allodynia at 6 days postoperative compared to the control (fibroblasts and PBS), but lack of long-lasting effects (< 2 days). Compared to the PBS control, pro-inflammatory fibroblasts induced transient hypersensitivity ipsilaterally in sham animals (*p < 0.05). **b** PBS and BMSC injection have no influence on the nociceptive behavioral of the contralateral hind paws, while fibroblast induces transient hypersensitivity (**p < 0.01). **c** Licking times after mechanical stimulation increased at days 4 post CCD surgery (^##^*p* < 0.01). **d** Sensorimotor coordination using the rotarod test shows no difference was observed between pre-injection and one day after second BMSC injection (day 4 and day 6). Each time point had a sample size of 9, **p < 0.01 compared with CCD + PBS control; *p < 0.05 compared with sham + PBS control. ^##^*p* < 0.01 compared with sham control

Thermal hyperalgesia and mechanical allodynia act through different signaling systems. To simplify our experimental model, we sought to determine the effect of BMSC on mechanical allodynia. Tactile allodynia, a form of mechanical allodynia and a hallmark of neuropathic pain, is defined as abnormal pain hypersensitivity evoked by innocuous stimuli [2]. To validate the effect of i.t. administration of BMSC on neuropathic pain in our CCD model, we employed a quantitative behavior test by enumerating hind paw withdrawals elicited by a defined innocuous mechanical stimulus (12 g von Frey filaments) as a measure for the severity of tactile allodynia. First, results showed that CCD, but not sham control animals, exhibited mechanical allodynia in the injured limbs starting 4 days post CCD, with CCD animals recording significantly larger paw withdrawals than sham animals (CCD + PBS vs. sham + PBS, 14.67 ± 2.12 vs. 2.11 ± 1.36, *p* < 0.001, Fig. 2a). Furthermore, there was no significant recovery of mechanical allodynia within 28 days post-CCD compared to baseline (CCD + PBS vs. sham + PBS, 19.67 ± 2.72 vs. 0.00 ± 0.00, *p* < 0.001, Fig. 2a). In contrast, no change in mechanical allodynia was observed on the contralateral hind paws of CCD and shame animals at different time points (*p* > 0.05; Fig. 2b). We also evaluated pain hypersensitivity by quantifying the number of licks after mechanical stimulation, and similarly, the CCD group showed significantly higher allodynia behavior than the sham surgery group (sham vs. CCD, 9.33 ± 2.73 vs. 38.00 ± 3.85, *p* < 0.001; Fig. 2c). Altogether, these results suggest that our CCD model was executed with specific and precise surgical methods that reliably produces neuropathic pain and its hallmark symptom of allodynia.

We tested the role of BMSC in CCD rats from the onset of neuropathic pain at 4 days post CCD, in the same time window as the behavior test mentioned above. When compared with CCD mice that did not receive BMSC injections, those treated with i.t. BMSC had significantly attenuated allodynia behaviors one day after treatment (CCD + BMSC vs. CCD + PBS, 12.67 ± 2.40 vs. 20.33 ± 3.28, *p* < 0.001; Fig. 2a), which represents a 37.6% reduction (antiallodynic effect (%) = 100(paw withdrawals in PBS control – paw withdrawals in BMSC group)/(paw withdrawals in PBS control)). However, 3 days after injection of BMSC (8 days post-CCD), allodynia behavior increased to largely equivalent levels of PBS injections (*p* > 0.05; Fig. 2a). To confirm that i.t. BMSC treatment does not affect healthy nerves, we also evaluated allodynia behavior in the unaffected contralateral limb, and no significant changes were seen in the contralateral limb (*p* > 0.05; Fig. 2b). We also evaluated motor coordination using the accelerating rotarod test to eliminate the potential confounding effects in the motor system possibly introduced by BMSC graft. No changes in motor performance were observed between pre- and post-i.t. BMSC treatment (*p* > 0.05; Fig. 2d). Finally, since BMSC cultures are known to be contaminated with fibroblasts, we also tested the effect of isolated fibroblast injection on allodynia in CCD. Fibroblasts caused significant changes in allodynia response in the ipsilateral (sham + PBS vs. sham + fibro, 1.67 ± 0.1.32 vs. 4.22 ± 1.56, *p* < 0.05; Fig. 2a) and contralateral limbs (CCD + PBS vs. CCD + fibro, 0.33 ± 0.52 vs. 2.33 ± 1.03, *p* < 0.001; Fig. 2b). However, these effects were opposite of those caused by BMSC treatment, suggesting that fibroblasts are not responsible for the analgesic effects of BMSCs. Together, these results indicate that i.t. delivery of BMSC causes a sizeable and transient reduction of neuropathic pain without causing changes in normal sensory or motor function.

### Intrathecally injected BMSCs do not localize in the spinal cord parenchyma or DRG after injection, and act via releasing intracellular contents

**Fig. 3:**
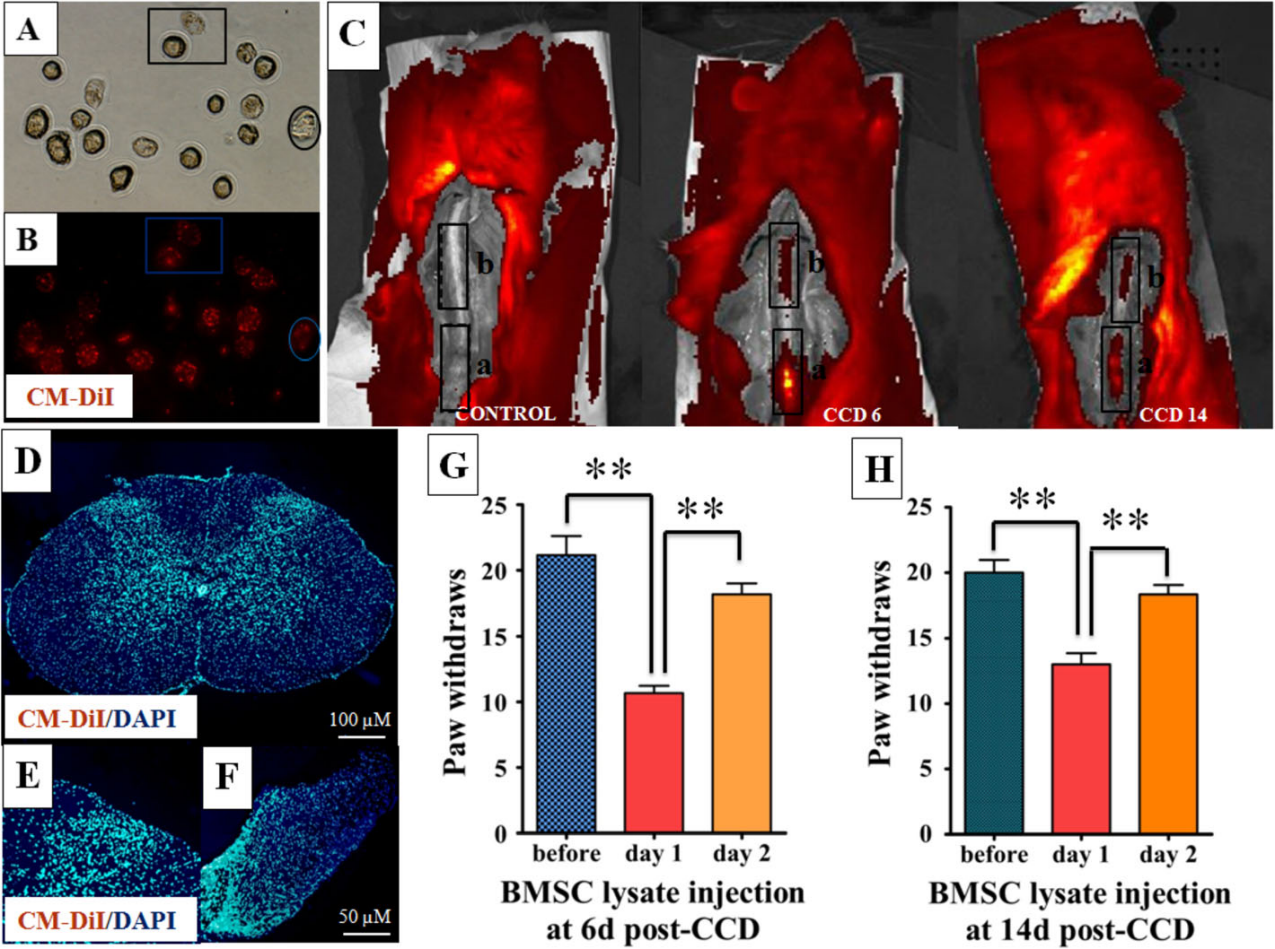
Mobilization of CM-DiI labeled BMSC by in vivo fluorescent imaging and cryosections. **a** Optical image of BMSC in a state of dissociation. **b** Fluorescent image of dissociated BMSC labeled with CM-DiI. Identical markings demarcate the same cells on optical images (**a**) and fluorescent image (**b**). **c** The spinal cord of BMSC-treated rats showed significant fluorescence at 6 and 14 days post-surgery. Rectangle **a** indicates the cavosurface of canalis spinalis which were removed caudally before imaging, and rectangle **b** indicates the lumbar enlargement. Pseudocolor images representing fluorescence signal intensity (crimson is the least intense and yellow is the most intense). **d**–**f** Cryosections of the spinal cord and DRG counterstained with DAPI for the nuclei. Grafted BMSC in the slices should be red and cell nuclei blue. **d** Full spinal cord section. **e** Photograph of the spinal dorsal horn. **f** Full DRG section. **g** Paw withdrawals decreased transiently when BMSC lysates were injected at day 6 post CCD. **h** Paw withdrawals also decreased transiently when BMSC lysates were injected at day 14 post CCD. **p < 0.01

To understand the mechanism of action of i.t. BMSCs, we evaluated whether i.t. BMSCs enter the spinal cord parenchyma and interact with DRG neurons. First, BMSCs were labeled by fluorescent stain and adherent BMSCs in culture dish were dissociated to increase staining efficiency. After dissociation, the cell bodies of BMSCs assumed circular profiles with large nuclei, and we confirmed that nearly all BMSCs were labeled (Fig. 3a, b). We then monitored the re-localization of BMSCs by in vivo fluorescent imaging and cryosections following i.t. BMSC administration. As expected, in vivo fluorescent imaging of PBS control rats showed no fluorescence in the spinal cord (Fig. 3c, left). In contrast, BMSC-treated rats showed significant fluorescence around the lumbar enlargement at 1-day post-injection, with the maximum fluorescence intensity located at the injection point on the cavosurface of canalis spinalis (Fig. 3c, middle). At 9 days post-injection (14 days post-surgery), fluorescence is more diffusively around the lumbar enlargement, and there was no apparent difference in fluorescence intensity (Fig. 3c, right). These results indicate that BMSCs were successfully transplanted to the lumbar spinal space. However, upon further evaluation, we found that there was no evidence of BMSCs in spinal cord parenchyma and DRG at 1 day (Fig. 3d–f) or 9 days (data not shown) post-injection. Grafted BMSCs in these slices would appear as red and cell nuclei are labeled blue. Together, these results suggest that i.t. BMSCs do not act locally and directly on spinal cord neurons, and there should be an intermediate step in its mechanism of action of reducing neuropathic pain, possibly via the release of BMSC’s intracellular contents.

To verify the hypothesis that the intracellular contents derived from BMSCs have meaningful effects on neuropathic pain, we injected BMSC-lysates intrathecally to the subarachnoid space of CCD animals at 6 days (early phase of neuropathic pain) and 14 days (late phase of neuropathic pain) post-CCD and assessed neuropathic pain-related behavior. One day after i.t. injection of BMSC-lysate for both time points, tactile allodynia was attenuated compared to pre-injection. The average paw withdrawals decreased by 49.7% (before vs. day 1, 21.17 ± 3.54 vs. 10.67 ± 1.37, *p* = 0.002; Fig. 3g) and 32.5% (before vs. day 1, 20.00 ± 2.37 vs. 13.50 ± 2.09, *p* = 0.002; Fig. 3h) for injection at 6-days and 14-days post-CCD, respectively. However, 2 days after injection at both time points, paw withdrawals of BMSC-lysate-treated animals returned to pre-injection levels (Fig. 3g, h). These results demonstrated that i.t. BMSC lysate was sufficient to reproduce the transient and robust analgesic effects of i.t. BMSC.

### Intrathecal BMSC does not affect TRPV4 expression on DRG neurons

**Fig. 4:**
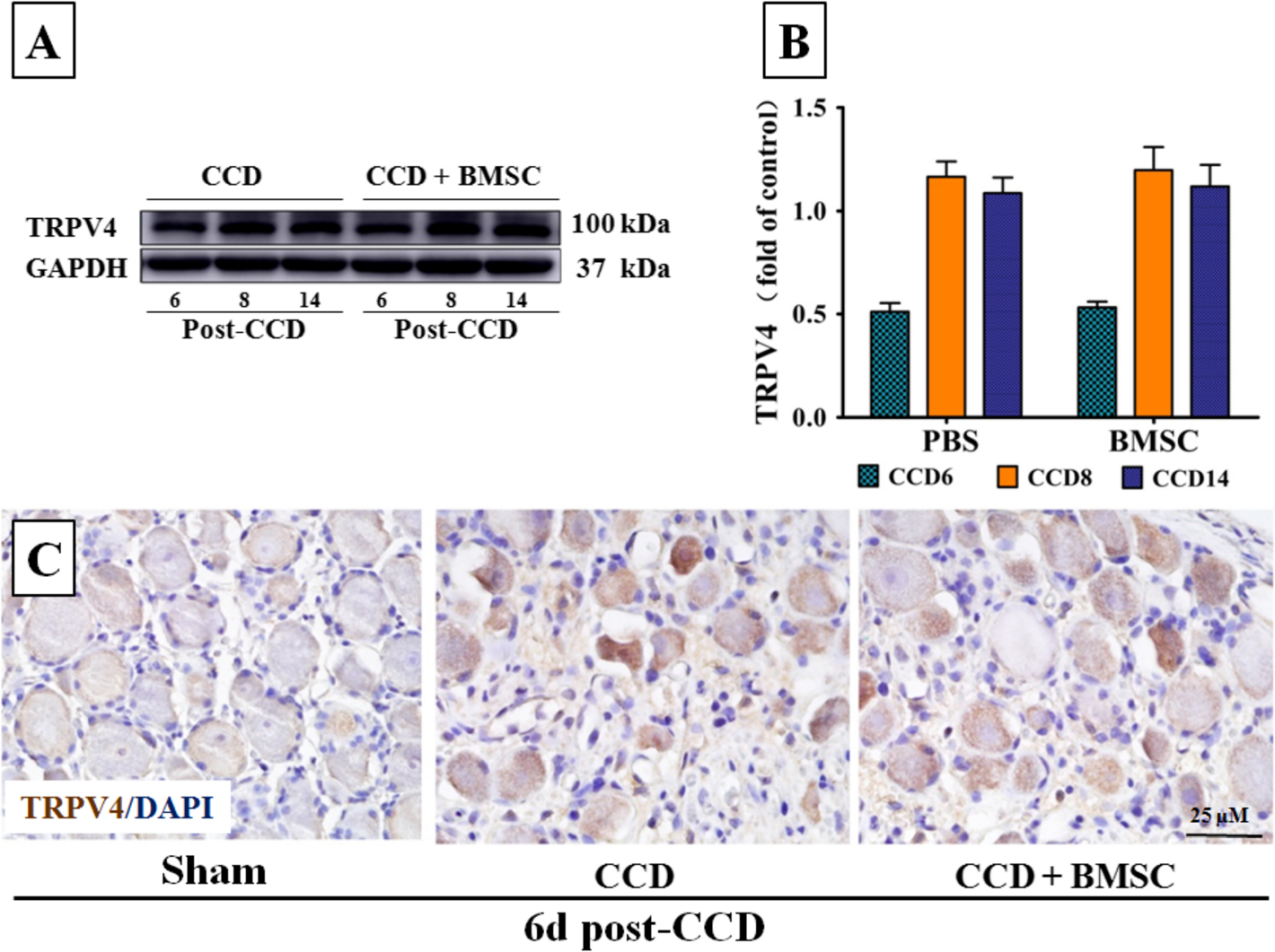
BMSC had no influence on the TRPV4 protein levels in ipsilateral DRGs. **a** Representative bands of TRPV4 protein from spinal cord ipsilaterally. **b** Western blot analysis of TRPV4 protein from the DRG ipsilaterally at different time points for CCD animals treated with i.t. PBS and BMSC. Band intensity was quantified as mean gray value and normalized to the control. GAPDH served as the loading control, each time point had a sample size of 6. **c** Immunohistochemistry results for TRPV4; BMSC graft has no effect on the immunoreactivity of TRPV4 in neurons of DRG

Dorsal root ganglia harbor sensory neuron somata and are thought to play a major role in the pathogenesis of pain. Researches from our group have demonstrated that TRPV4 in DRG neurons are upregulated following CCD in rats [30, 37]. To examine whether i.t. delivery of BMSCs acts via modulating TRPV4 in the DRG, we quantified TRPV4 protein expression at various time points. As shown in Fig. 4 a and b, BMSCs have no influence on the level of TRPV4 protein in CCD rats and similar results were obtained in immunohistological analyses at 6 days post CCD (Fig. 4c).

### Intrathecal BMSC attenuates neuropathic pain via microglial activity independent of microglial activation

**Fig. 5:**
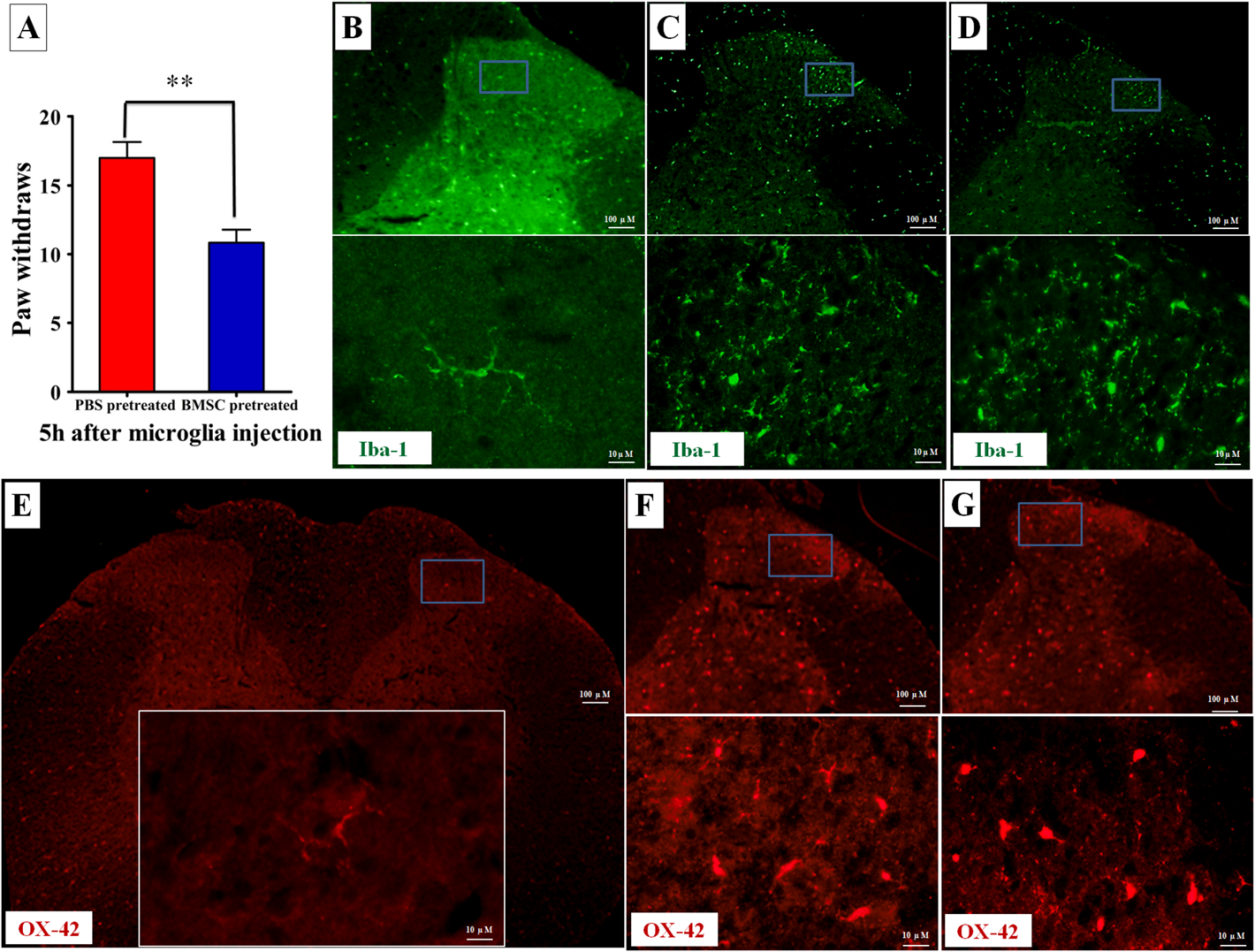
Microglia activation represented by Iba-1 and OX-42 immunofluorescence at 6 days post-CCD. **a** Compared to baseline, microglia (i.t.) pretreated with PBS and BMSC-lysate both induced tactile allodynia; BMSC-lysate pretreated microglia triggered fewer paw withdrawals compared with PBS pretreated microglia. **b** Iba-1 signal revealed moderate expression of resident microglia in both white and gray matter of the lumbar dorsal horn in sham rats; microglia exhibit a resting type morphology (inset). **c** Six days after CCD, microglia Iba-1 immunofluorescence increased significantly, and microglia exhibited activated phenotype (inset). The superficial laminae of the spinal dorsal horn (demarcated with green line) shows that microglia are a dominant population, consistent with their important role in nociceptive transmission and modulation. **d** Iba-1 immunoreactivity in the spinal cords was not altered by BMSC-treatment compared to the CCD control. **e**–**g** OX-42 signal also revealed that BMSC graft has no effect on microglia activation; **e** a resting type morphology (inset), **f** the dorsal horn from CCD animals, and **g** the dorsal horn from BMSC graft animals. **p < 0.01

Spinal microglia play a key role in the pathological and chronic pain mechanisms, and microglia activation has been implicated in the onset and maintenance of neuropathic pain following peripheral nerve or spinal cord injury [17, 18, 20]. Past studies have shown that intraspinal administration of ATP-stimulated microglia to naïve and uninjured rats is sufficient to produce allodynia and neuropathic pain [22, 25, 38]. To determine whether microglia are involved in the anti-allodynic effect of BMSC, we cultured microglia treated with or without BMSC lysate, stimulated them with ATP, and injected the microglia (1000 cells/10 μL) intrathecally to the lumbar spinal space of naïve, uninjured rats. Neuropathic pain was assessed 5 h after injection. We found that paw withdrawals elicited by innocuous mechanical stimulus markedly increased 5 h after i.t. delivery of ATP-stimulated microglia without BMSC lysate treatment. This confirms that ATP-stimulated microglia are able to generate neuropathic pain. In contrast, injection of ATP-stimulated microglia pretreated with BMSC lysate resulted in significantly fewer paw withdrawals (PBS vs. BMSC pretreated, 17.00 ± 2.83 vs. 10.83 ± 2.31, *p* = 0.002; Fig. 5a). These results suggest that tactile allodynia in response to ATP-stimulated microglia was partially negated by BMSC lysate.

To further explore the possible effects of BMSCs on microglia, we examined whether i.t. BMSC had an effect on the activation of microglia following nerve injury. We performed an analysis of ionized calcium-binding adapter molecule 1 (Iba-1) and OX42 (CD11b/c equivalent protein) immunofluorescence in the lumbar spinal cord, which are features of microglia activation. In sham animals, Iba-1-positive cells demonstrated small compact somata and slender branched processes (Fig. 5b), consistent with the resting type morphology of microglia. Six days after CCD, a prominent increase in Iba-1 immunostaining in the ipsilateral dorsal horn was observed. These microglial cells exhibited an activated phenotype, with cellular hypertrophy and retraction of cytoplasmic processes (Fig. 5c). BMSC transplantation had no significant effect on Iba-1 immunoreactivity and the number of microglia when compared with tissue from CCD animals without treatment 6 days post-surgery (Fig. 5d). Similar results were also obtained in the OX42 immunofluorescence of the spinal cord slices (Fig. 5e–g). Together, these results imply that microglia activation is associated with neuropathic pain following CCD surgery, but i.t. BMSC did not have an impact on microglial activation.

Together, these results suggested that while BMSC did not modify microglial activation, it is intracellular contents may curtail the role of activated microglia in the pathogenesis of neuropathic pain. Thus, the regulation of glial signaling molecules or glial mediators rather than glial activation per se may be more central to the mechanism of i.t. BMSC therapy.

### BMSCs attenuate the expression of P2X_4_R protein in activated spinal microglia after CCD and reduce microglial P2X_4_R upregulation in response to fibronectin

**Fig. 6:**
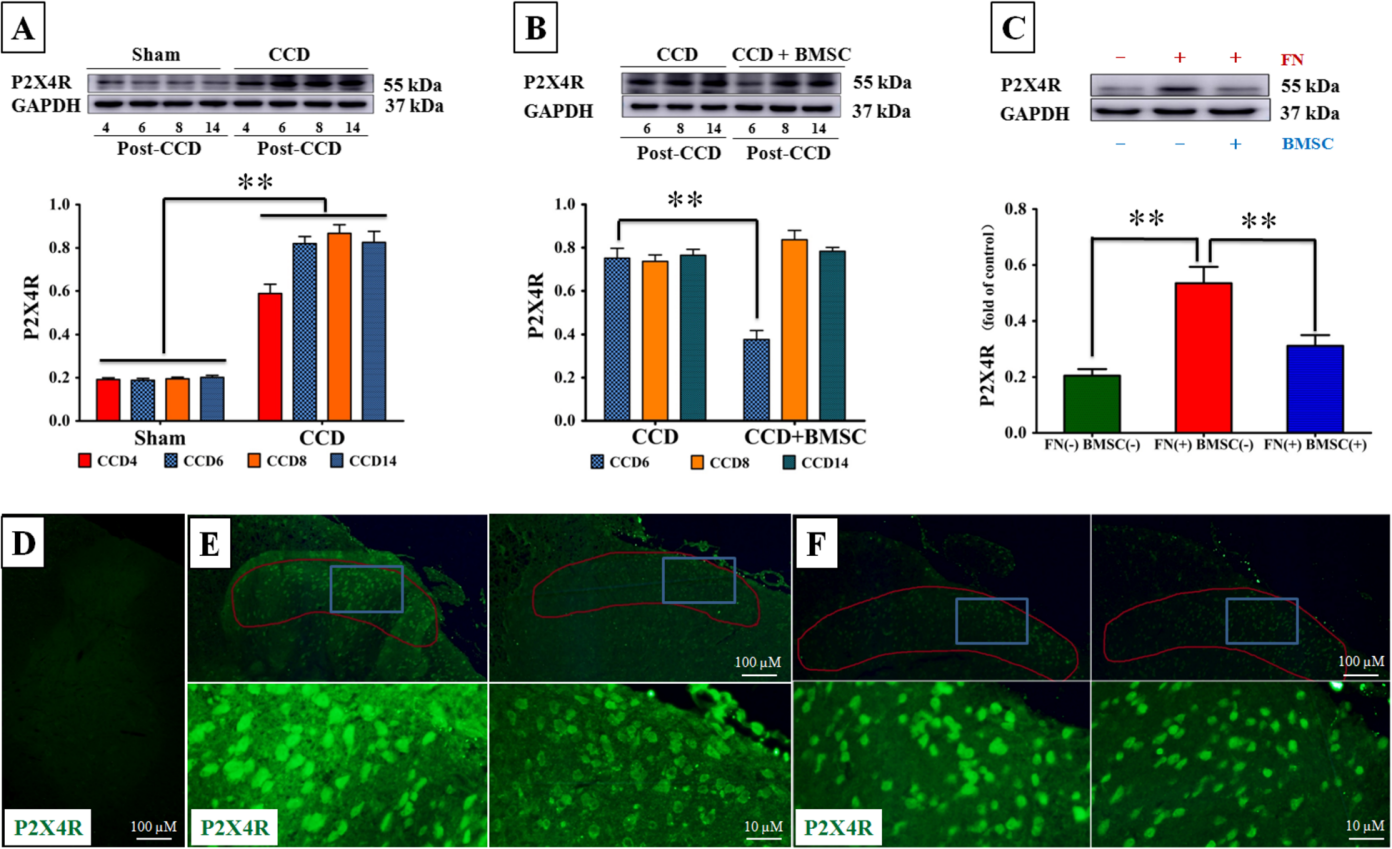
Intrathecal BMSCs attenuate the upregulation of P2X_4_R provoked by CCD. **a** Western blot analysis of P2X_4_R proteins in the membrane fraction from the spinal cord ipsilateral to surgery at different time points in sham and CCD rats. **b** BMSC injection downregulated the level of P2X_4_R protein at days 6 post-CCD. **c** Primary microglia were incubated with BMSC lysate for 24 h, and the upregulation of P2X_4_R expression induced by FN (10 μg/mL) was suppressed. GAPDH served as a loading control. Each time point had a sample size of 6 and data are presented as the mean percentage of each control (SEM). **d** P2X_4_R immunofluorescence was barely detectable in sham animals. **e** At 6 days post-CCD, P2X_4_R immunoreactivity was clearly induced in the dorsal horn ipsilateral to the lesion side (left top and left bottom); this signal was weaker in the lumbar spinal cords from BMSC graft animals (right top and right bottom). Of note, microglia remain the dominant population in the dorsal horn for both groups (demarcated by the red line), suggesting that BMSC treatment did not affect the number of microglia residing in this area. **f** At 14 days post-CCD, there was no difference in the P2X_4_R immunoreactivity between the CCD rats (left top and bottom) and BMSC-treated (right top and bottom). Bottom photography in **e** and **f** are magnified images of their corresponding parent image above (area magnified demarcated by a blue square). **p < 0.01

Upregulation of P2X_4_R, a protein expressed exclusively in microglia [25], is known to contribute significantly to the genesis of tactile allodynia after periphery nerve injury [24, 25, 38]. To investigate the detailed mechanism by which BMSC curtails pain-generating functions of activated microglia, we analyzed P2X_4_R expression in the spinal cord after CCD using immunoblot and immunohistochemistry. Using immunoblot studies, we found that the level of P2X_4_R proteins from the ipsilateral spinal cord of CCD animals is elevated from 4 to 14 days post-operation compared with sham controls (*p* < 0.001, Fig. 6a). We also found that the level of P2X_4_R proteins in homogenates from the spinal cord of BMSC-treated animals was significantly decreased than that of CCD animals; here, the effects of BMSCs on P2X4R expression were only observed on post-operation day 6 (CCD vs. CCD + BMSC, 75.17 ± 4.53% vs. 37.67 ± 4.14%, *p* < 0.001l; Fig. 6b), which coincides with the analgesic effects of BMSC transplantation. To further confirm that BMSC-derived factors are also able to curtail microglia P2X_4_R expression, P2X_4_R immunoblot was investigated in microglia primary cultures. Microglia cells were plated on dishes coated with fibronectin (FN, 10 μg/mL), a factor implicated in P2X_4_R upregulation in microglia and tactile allodynia [23]. In microglia cells incubated with BMSC lysate, an increase in the P2X_4_R protein level by fibronectin was markedly suppressed (FN(+) BMSC(−) vs. FN(+) BMSC(+), 53.50 ± 14.36% vs. 29.17 ± 7.13%, *p* = 0.001; Fig. 6c).

Our immunohistochemistry data also demonstrated consistent results. Six days after CCD, we observed strong, punctate P2X_4_R immunofluorescence dotted throughout spinal dorsal horns of CCD animals (Fig. 6e, left) which were absent in sham control animals (Fig. 6d). After i.t. injections of BMSC, P2X_4_R immuno-reactivity was weaker than in untreated CCD animals at CCD6 but not at CCD14 (Fig. 6e, f). Collectively, these results strongly suggest that BMSCs cause reduced activation of P2X_4_R in spinal microglia after CCD and downregulation of P2X_4_R in response to fibronectin in vitro.

### Inhibiting microglial P2X_4_R is necessary and responsible for i.t. BMSC’s analgesic effect

**Fig. 7:**
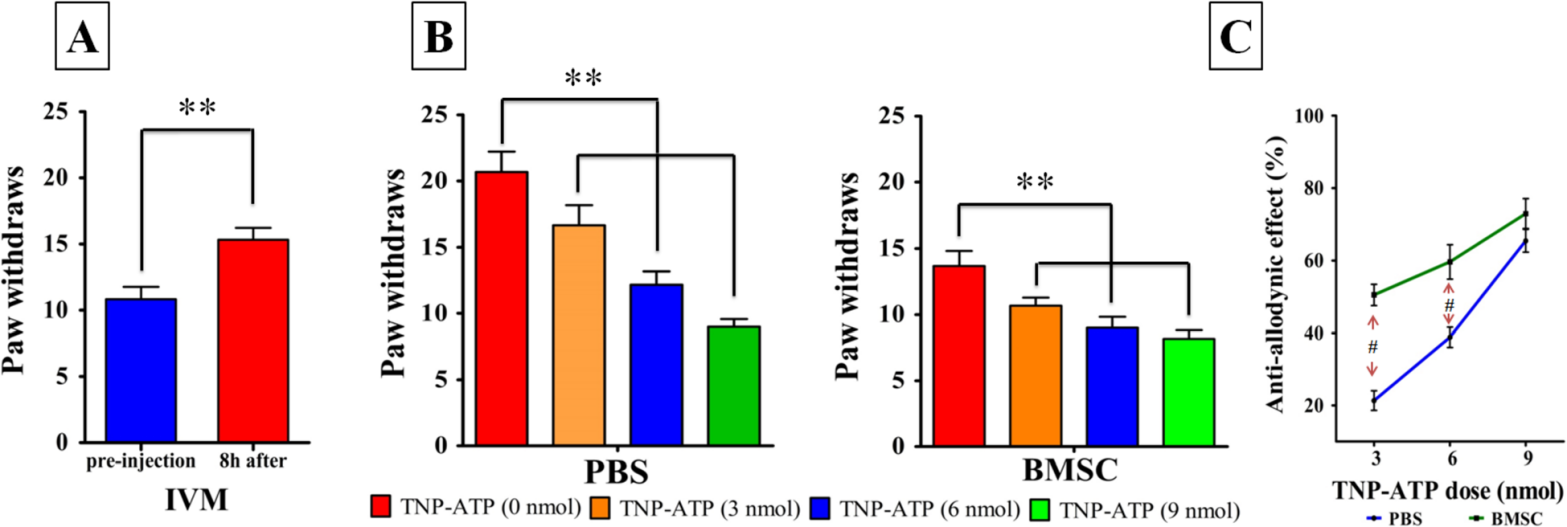
Mechanistic link between P2X_4_R and anti-allodynic effect. **a**–**c** Pharmacological interventions of P2X_4_R. **a** After 8 h, IVM (positive allosteric modulator of P2X_4_R subtype) reduces the therapeutic effect of BMSC graft. **b** TNP-ATP (antagonist of P2X_4_R subtype) caused the decline of paw withdrawals both in CCD and CCD + BMSC animals in a dose-dependent manner. **c** Anti-allodynic effect between the two groups with increasing TNP-ATP doses; anti-allodynic effect (percentage) was defined as 100% *(paw withdrawals in PBS control – paw withdrawals after injection)/(paw withdrawals in PBS control). Each time point had a sample size of 6, **p < 0.01, ^#^*p* < 0.05 compared with PBS control

Although BMSC-induced downregulation of P2X_4_R coincided with BMSC-induced analgesia, whether modulation of P2X_4_R was required for i.t. BMSC’s analgesic effect remained unclear. To address this question, we injected ivermectin (IVM, a positive allosteric modulator of P2X_4_R) intraperitoneally to BMSC treated CCD animals 6 days postoperatively and assessed allodynia. Our results revealed that ivermectin reduced the analgesic effects of i.t. BMSC treatment (pre-injection vs. 8 h after, 15.33 ± 2.16 vs. 10.83 ± 2.31, *p* = 0.007; Fig. 7a). To further evaluate whether P2X_4_R downregulation is responsible for i.t. BMSC analgesia, we also examined whether TNP-ATP (an antagonist of P2X_4_R) can reduce the marginal benefit of i.t. BMSC compared with control. Our results showed that TNP-ATP caused the decline of paw withdrawals in CCD (13.33 ± 2.53 vs. 20.83 ± 2.47 at 6 nmol TNP-ATP, *p* = 0.002; Fig. 7b, left) and CCD + BMSC animals (14.15 ± 2.16 vs. 8.91 ± 2.04 at 6 nmol TNP-ATP , *p* = 0.006; Fig. 7b, right ). With increasing doses of TNP-ATP, the differences in analgesia between CCD animals with and without i.t. BMSC treatment decreased (*p* = 0.02; Fig. 7c), suggesting that i.t. BMSC supplies a competitive antagonist of P2X_4_R that may share a similar binding site with TNP-ATP. Together, these findings suggest that modulation of P2X_4_R is required for i.t. BMSC’s analgesic effects, and that this effect is likely achieved via the release of the competitive antagonist(s) that likely binds to a similar site on P2X_4_R as TNP-ATP.

## Discussion

Neuropathic pain is a substantial clinical problem worldwide and currently lacks effective treatments. In this study, we found that i.t. delivery of BMSCs is efficacious for short-term alleviation of neuropathic pain at 6 days post-injury, and that this therapy’s effect is largely mediated by the release of BMSC intracellular contents into the i.t. space leading to a downregulation of microglial P2X_4_R expression. Neuropathic pain can be roughly divided into the early phase (less than 7 days post-injury) and the late phase [39], and our results serve as an expansion of foundational understanding of using i.t. BMSC therapy as a safe and effective treatment for early-phase neuropathic pain. This strategy also holds significant potential for clinical translation, as i.t. delivery of BMSCs features a favorable safety profile and MSCs are easily accessible and can expand to clinical scales in a relatively short period of time. Our novel insights regarding the mechanisms of BMSC therapy can further guide the optimization of i.t. BMSC therapy for clinical translation.

Biosafety is a critical concern that has limited the development of BMSC therapy for neuropathic pain. While immunosuppressive properties of BMSCs can ameliorate neuronal damage in various central nervous system diseases, including chronic neuropathic pain [8, 11, 40], recent studies highlighted many risks associated with systemic BMSC therapy particularly in immuno-compromised conditions, including potential maldifferentiation, immunosuppression, and instigation of malignant tumor growth [41–43]. In this study, we confirmed that i.t. delivery of BMSC has a favorable safety profile. We did not observe adverse events, including influence on normal nociceptive behavior. We also did not observe any tumorigenesis in the spinal cord for up to 28 days post-CCD. Thus, our data suggest that i.t. BMSC poses minimal health risks, and that targeted intrathecal delivery of BMSCs may be more preferable for treating neuropathic pain than other administration strategies.

While the effectiveness of i.t. BMSC injection in neuropathic pain is debatable, our data suggest that i.t. BMSC delivers a transient yet sizeable attenuation of pain hypersensitivity in CCD rats. To explore the mechanisms of this effect, we first sought to decipher where (anatomically) i.t. BMSCs are acting in our model. The mechanism of BMSC’s efficacy in neuropathic pain was previously thought to be contact-dependent; however, soluble factors may also play a key role in the induction of BMSC-mediated effects. Surprisingly, we did not find evidence of viable BMSCs in the DRG or spinal cord. This is in contrast to Chen et al., who previously reported that i.t. BMSCs migrate from the injection site to the border of DRGs and remain viable for more than 2 months to deliver long-lasting analgesic effects [9]. The viability of injected stem cells has been an area of controversy; prior studies have also shown that grafted MSCs were restricted to the injection site with no migration [14] and that only a small amount of BMSCs could be found in the anterior spinal artery [15]. These discrepancies may have arisen from differences in the experimental set up (e.g., culture conditions [44] or BMSC sources), inherent heterogeneity of BMSC’s biology [45], animal model difference, and sex-specific differences. To account for heterogeneity of BMSC’s biology, characterization of BMSCs is a critical first step. In our study, we used commercially validated cells that express CD29, CD44, CD90, and CD106, and are negative for CD11b, CD34, and CD45, without further characterization. We did, however, control for fibroblast contamination (which is a known phenomenon with BMSC cultures) in our animal experiments by including isolated fibroblast injection as a separate group. Next, animal model differences, such as the degree of injury and host immunoreaction, may also play a major role in BMSC survival and tissue integration of BMSCs [46]. In the present study, we employed the CCD model, which, compared to other neuropathic pain models, induces a longer-lasting structural and functional changes throughout the nociceptive pathway via establishing persistent intervertebral foramen stenosis. Thus, it is possible that intrathecally injected BMSCs in CCD rats may have reduced viability due to a stronger inflammatory response in the CCD model, and the lack of nutritional support in the spinal fluid may further facilitate inevitable apoptosis of i.t. BMSCs. Despite our findings showing the absence of BMSCs in the DRG or spinal cord, we were able to observe a strong analgesic effect. Together, these findings suggest that the analgesic effect of intrathecally injected BMSCs does not necessitate cellular contact, which provides novel insight into BMSC’s mechanism of action on alleviating neuropathic pain. Furthermore, given the transient nature of i.t. BMSC’s analgesic effects and its favorable safety profile compared to other routes of administration, multiple i.t. injections, though not be optimal due to potentially more damage to the surrounding tissue of the spinal cord, might have a stronger impact on neuropathic pain. Future research is warranted to explore and evaluate the effectiveness of multiple i.t. BMSC injections. Finally, research has shown that endogenous sex steroids play a key role in mediating differences in nociception [47]. Animals used in the present investigation are male, and further investigation is needed to elucidate potential sex-specific differences for response to i.t. BMSC treatment in neuropathic pain.

Interestingly, our findings of lack of BMSCs in the spinal parenchyma and DRG suggest that i.t. BMSCs are not homing to nociceptive neurons. While the lack of BMSC homing may contribute to the transient nature of analgesic effects, these findings also suggest that homing is not necessary to deliver significant analgesia, at least in the short term. Thus, we hypothesized that soluble factors released from BMSC are responsible for the attenuation of pain hypersensitivity following i.t. BMSC therapy. We verified this hypothesis by injection of BMSC lysates (2 million/15 μl) to the subarachnoid space at 6 and 14 days post-CCD. Our results demonstrated that BMSC lysates retarded neuropathic pain behavior to a similar extent as seen in i.t. BMSC-treated animals. Past studies have suggested that injection of molecules secreted by BMSCs may be more effective than transplanting whole cells in certain disease states [48]. MSCs are known to secrete a broad range of neuromodulatory factors at very low levels [49]. Further enhancement of i.t. BMSC-based therapy can be achieved by isolating these critical components and administering them alone which may be an effective strategy to treat neuropathic pain. Another possible strategy to augment BMSC-based therapy may be achieved by incorporating genetic engineering, where expression of therapeutic peptides in BMSCs (e.g., glia-derived neuropathic factor (GDNF), IL-10, TGF-β) can be increased as much as 2000-fold via viral transduction [14, 50], thus enhancing the delivery of these potentially therapeutic peptides to treat neuropathic pain [14]. Overall, our findings reveal a previously unknown ability of BMSCs to deliver analgesic effects that is independent of homing to nociceptive and cellular contact; this new may be a valuable mechanistic insight for future optimizations of neuropathic pain therapies. While we believe that soluble factors released from BMSCs in our model may be responsible for transient analgesia, studies specifically investigating homing markers such as CXCR4 and CXCL12 are needed to further elucidate underlying mechanisms.

To decipher the cellular or molecular targets of i.t. BMSC therapy, we first investigated whether DRG neurons are affected. TRPV4 is a crucial mechani- or osmo-receptor expressed on DRG neurons, and the levels of TRPV4 mRNA and protein are upregulated after CCD, suggesting that TRPV4 plays an important role in neuropathic pain [30, 37]. Our results showed that the upregulation of TRPV4 in the DRG was not influenced by i.t. delivery of BMSC. This lack of effect may be due to the following two reasons: (1) DRG somas are not in direct contact with CSF constituents, and soluble factors relinquished from BMSCs may not easily cross the blood-spinal cord barrier, and (2) with persistent intervertebral foramen stenosis in CCD, the upregulation of TRPV4 may be too severe to be modified by only modest amounts of molecular contents relinquished by BMSCs.

The immune system plays a crucial role in neuropathic pain, and over the past several years, dedicated works from several groups have demonstrated key interactions between BMSCs and the innate and adaptive immune systems [51–53]. Clinically, BMSC’s immunosuppressive ability was first validated through successful treatment of severe acute graft-versus-host disease that was refractory to steroid immunosuppression [54]. Upon CNS injury, microglia rapidly respond to adverse physiological conditions and switch to an activated state, thus producing pro-inflammatory (neurotoxic) and neurotrophic (neuroprotective) factors upon activation of various cell-surface receptors [55]. In this study, using an in vivo microglia transfer approach, we clearly demonstrate microglial involvement in the analgesic effect of BMSC. However, our data suggest that the upregulation of microglial marker Iba-1 and OX42 was not mitigated by BMSC administration despite BMSC’s analgesic effects. This result suggests that microglial activation may not directly modulate pain hypersensitivity, which may seem to contradict the current dogma. However, microglia activation is a complex process, and microglia are known to have different functional states, each exhibiting pro-inflammatory (neurotoxic, M1) or anti-inflammatory (neuroprotective, M2) phenotypes [56, 57]. Further investigation is needed to elucidate the role of microglia activation in i.t. BMSC.

Past studies have identified purinoceptors as key players in bidirectional communications between microglia and other cells in the nervous system, which control several properties of microglia including the motility of their fine processes, the release of cytokines, their migration, and their phagocytosis [26]. The upregulation of P2X_4_R in spinal microglia after peripheral nerve injury is critical for the pathogenesis of pain hypersensitivity after nerve injury [24, 25, 38]. In line with previous studies, our experiments showed that CCD induces an upregulation of P2X_4_R expression in the spinal cord ipsilateral to the lesion. We then investigated the effect of i.t. BMSC on microglial P2X_4_R expression. For the first time, we showed that expression levels of microglia P2X_4_R protein provoked by CCD in the spinal cord and induced by fibronectin in primary cultures are attenuated by BMSC graft and BMSC lysate, respectively. We further verified the direct involvement of P2X_4_R modulation in the alleviation of pain hyperalgesia using pharmacological interventions. At the time when peak analgesic effect was observed, administration of IVM (positive allosteric modulator of P2X_4_R) retarded the therapeutic effect of BMSC. Meanwhile, TNP-ATP (antagonist of P2X_4_R) induced a significant decrease in the hind paw withdrawals to innocuous stimuli. Furthermore, the efficiency of anti-allodynia of TNP-ATP in PBS control was close to that in BMSC-treated animals at high TNP-ATP doses, suggesting that BMSC derived factors may be acting on the same binding site as TNP-ATP. While our results suggest that alteration of P2X_4_R expression is a major mechanism of action of i.t. BMSCs, P2X_4_R may not be the only target of BMSC derived factors, and it is conceivable that, in addition to its effect on microglia, BMSCs could suppress neuropathic pain by modulating multiple processes in a combinatorial fashion [58, 59]. As future BMSC-based therapeutics are developed for neuropathic pain, we believe that combination therapies directed at multiple targets in neuropathic pain might be worthwhile. Although our experimental therapy may not perfectly serve as proof-of-concept, our identification of microglial P2X4R downregulation as a mechanistic component of i.t. BMSCs provides a valuable addition to the current literature and knowledge of i.t. BMSC therapy in neuropathic pain. Further research is now eagerly awaited to elucidate the mechanisms underlying the downregulation of P2X_4_R by BMSC and its cross-talk with other analgesic pathways.

## Summary and conclusions

Overall, our results demonstrate that intrathecal BMSC injection is a safe and effective approach for the treatment of neuropathic pain after nerve injury and provides transient yet significant analgesia. Our mechanistic investigations suggest that these effects are likely due to the release of soluble cellular contents and their subsequent inhibition of microglial P2X_4_R, and not due to BMSC differentiation or contact-dependent modulations of DRG neurons. These new findings offer valuable insight into the mechanism of BMSC therapy in the context of neuropathic pain and open new avenues for further investigation and optimization.

## Data Availability

All data supporting the conclusions of this manuscript are provided in the text and figures. Please contact the author for data requests.

## Abbreviations

BMSC: Bone marrow stromal cells
CCD: Chronic compression of dorsal root ganglion
CSF: Cerebrospinal fluid
DRG: Dorsal root ganglion
FN: Fibronectin
i.t.: Intrathecal
Iba-1: Ionized calcium-binding adapter molecule 1
IVM: Ivermectin
P2X4R: Purinergic receptor P2X4
TRPV4: Transient receptor potential vanilloid 4
